# Iguratimod in Autoimmune Diseases: Emerging Evidence for its Immunomodulatory and Therapeutic Role

**DOI:** 10.31138/mjr.060925.iar

**Published:** 2026-05-20

**Authors:** Clara Joseph Shilpa, Nikita Chettri, Hema Sameera Pinnam, Sruthy Jose, Mahabaleshwar Mamadapur

**Affiliations:** 1 Department of Pharmacy Practice, JSS College of Pharmacy, Mysuru, JSSAHER, Mysuru, Karnataka, India;; 2 Department of General Medicine, JSS Medical College and Hospital, JSS Academy of Higher Education, Mysore, Karnataka, India;; 3 Department of Clinical Immunology and Rheumatology, JSS Medical College and Hospital, JSS Academy of Higher Education, Mysore, Karnataka, India

**Keywords:** ankylosing spondylitis, autoimmune disease, NF-Kappa B, antirheumatic agents

## Abstract

**Background:**

Rheumatoid arthritis (RA) is a chronic autoimmune disorder marked by joint inflammation and damage, leading to disability. Traditional treatments include methotrexate and other DMARDs, but many patients experience inadequate responses or intolerable side effects. Iguratimod (IGU), a novel synthetic DMARD developed in Japan, has shown promise in the management of RA and related autoimmune conditions.

**Methodology:**

A comprehensive literature review was conducted using databases such as PubMed, Google Scholar, ScienceDirect, Web of Science, and Medline, focusing on recent high-quality publications related to iguratimod and employing keywords like “Iguratimod,” “Sjögren’s syndrome,” “Sjögren’s disease”, “Rheumatoid arthritis,” and “Ankylosing spondylitis”.

**Results and Discussion:**

Clinical trials have demonstrated that iguratimod effectively reduces disease activity in RA patients, showing a comparable efficacy to salazosulphapyridine and improved outcomes when combined with methotrexate. In patients with Sjögren’s disease, IGU significantly decreased disease activity indices and immunoglobulin levels. Furthermore, studies indicate that IGU has a favourable safety profile with fewer adverse effects than traditional DMARDs. The findings suggest that IGU offers a viable alternative for patients with RA and other autoimmune diseases who are intolerant to conventional therapies. Its dual action as an immunomodulator and anti-inflammatory agent positions it as a beneficial option for managing these chronic conditions.

**Conclusion:**

Iguratimod is a promising therapeutic option for the treatment of rheumatoid arthritis, Sjögren’s disease, and ankylosing spondylitis. Further research is warranted to establish long-term efficacy and safety profiles and explore its potential in combination therapies for patients with inadequate responses to existing treatments.

## INTRODUCTION

**Figure 1.**
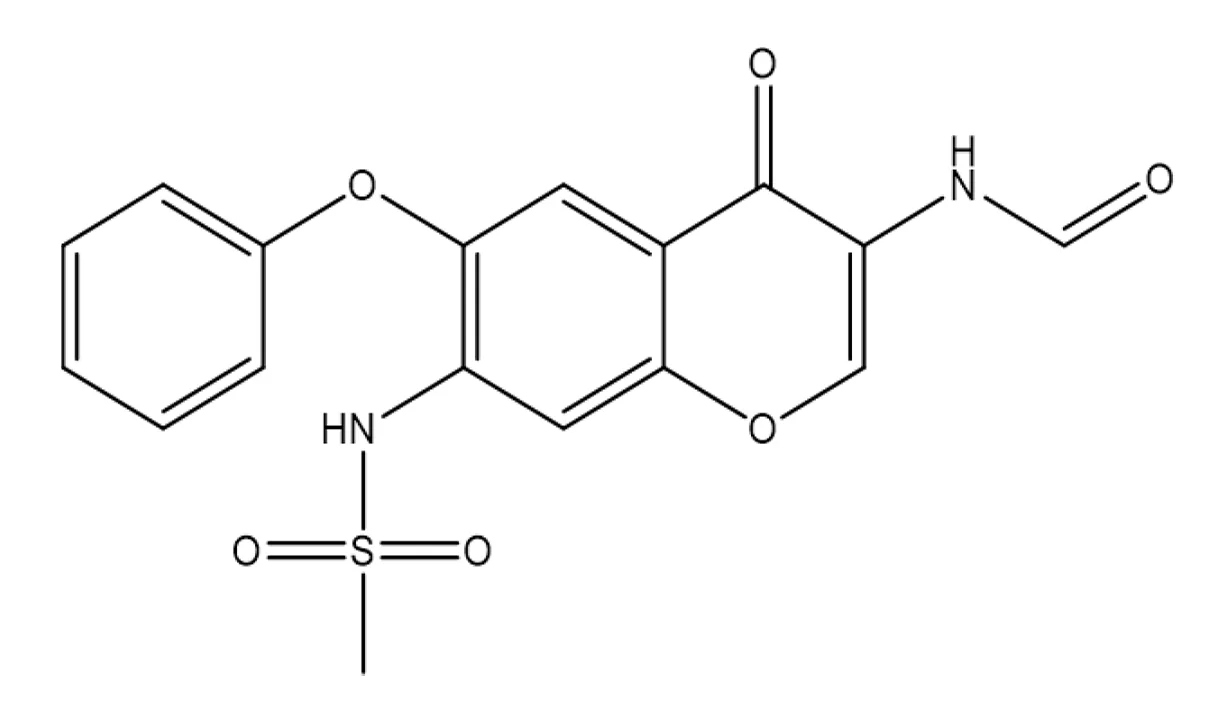
Chemical structure of iguratimod (ChemDraw).

Rheumatoid arthritis (RA) is a chronic autoimmune disorder with an unknown cause, characterised by joint synovitis, progressive bone damage, and loss of joint functionality.^1 1^The treatment of RA has primarily relied on methotrexate and other disease-modifying anti-rheumatic drugs (DMARDs), whether used alone or in combination.^2^ Nevertheless, the rate of remission among RA patients continues to be low.^3^ Iguratimod is a methane sulfonanilide that is chemically composed of (N-[7-[( methanesulfonyle ) amino ] −4-oxo-6-phenoxy-4H-1-benzopyran-3-yl]-formamide) (**Figure 1)**.

This drug is a novel synthetic small-molecule DMARD developed in Japan.^3,4^ Additionally, Iguratimod has demonstrated effectiveness in treating Sjögren’s disease (SjD), a chronic inflammatory autoimmune disease primarily affecting exocrine glands, particularly the salivary and lacrimal glands, and often associated with other chronic autoimmune diseases involving visceral organs.^1,3,5^

## MODE OF ACTION

**Figure 2.**
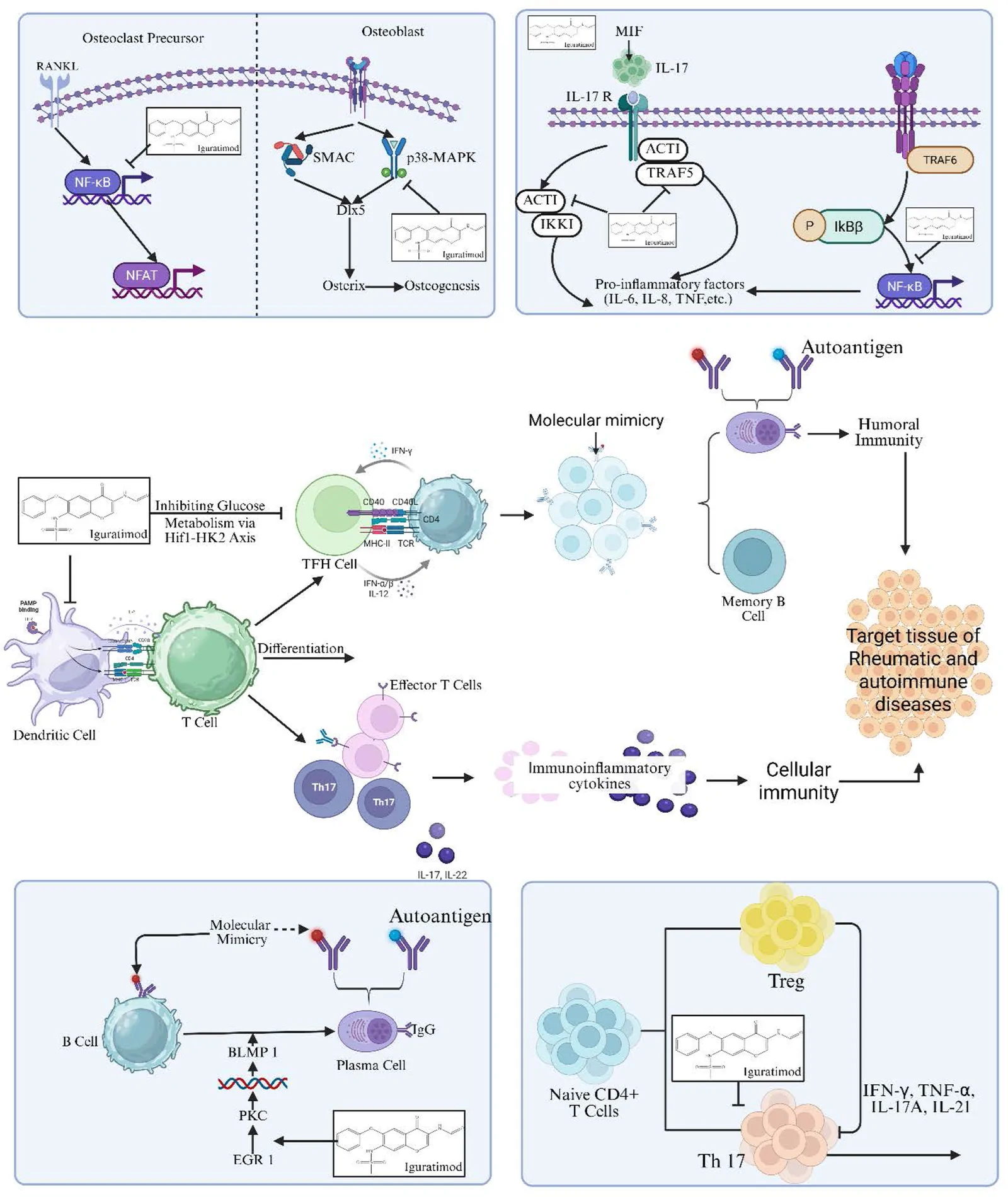
Regulatory mechanism of iguratimod on inflammatory signalling pathways.

Iguratimod has been identified as an inhibitor of interleukin (IL) −1 and IL-6 expression in monocytes. It selectively reduces cyclooxygenase (COX) −2 activity and decreases COX-2 mRNA levels. Additionally, IGU can inhibit nuclear factor-kappa B (NF-kB) activation by preventing its translocation from the cytoplasm to the nucleus, without affecting the degradation of Inhibitor of kappa B alpha (IκBα) in THP-1 cells stimulated with lipopolysaccharide. Furthermore, it suppresses IgM and IgG expression in B cells, reducing immunoglobulin production through direct effects on B lymphocytes, without affecting their proliferation, in patients with rheumatoid arthritis. IGU influences cellular immunity by downregulating inflammatory cytokines and transcription factors associated with Th1, Th17, and follicular helper T cells while simultaneously upregulating cytokines and transcription factors linked to regulatory T cells.^1,3, 6^ Given the significant roles of T and B cells in the pathogenesis of Sjögren’s disease, Iguratimod is considered a promising treatment option for this condition.^5^ Iguratimod effectively inhibits the production of pro-inflammatory cytokines such as tumour necrosis factor-alpha (TNF-α), IL-1, IL-6, IL-8, and IL-17 by blocking the activation of nuclear factor-kappa B (NF-κB) in cultured human synovial cells and human acute monocytic leukaemia cells.^3,7^ Additionally, it facilitates bone resorption by promoting osteoblast differentiation and inhibiting osteoclast differentiation in RAW264.7 cells, thereby protecting against bone destruction.^4,6,8^ As a result, Iguratimod is considered an effective treatment for Ankylosing Spondylitis (AS), a chronic inflammatory autoimmune disease that mainly affects the axial joints.^2,9^ The drug can also inhibit NF-kB nuclear trans-location and mitigate associated pro-inflammatory responses. Furthermore, it has been found to reduce the phosphorylation of Mitogen-Activated Protein Kinases (MAPKs) and to target Act1, thereby disrupting its interaction with TRAF5 or IKKi in the IL-17 signalling pathways in synoviocytes.^3^ Additionally, Iguratimod significantly lowers the expression levels of matrix metalloproteinases MMP-1 and MMP-3 in synovial fibroblasts from rheumatoid arthritis patients.^8^ A schematic representation of Iguratimod’s regulatory effects on key signalling pathways is shown in **Figure 2**.

In summary, iguratimod functions as a unique, small-molecule DMARD with a multifaceted mechanism of action. It integrates direct immunomodulation—by suppressing B-cell immunoglobulin production and rebalancing T-cell subsets—with potent anti-inflammatory effects achieved through inhibition of NF-κB and MAPK signalling, leading to reduced levels of pro-inflammatory cytokines (IL-1, IL-6, TNF-α, IL-17). Furthermore, its favourable impact on bone metabolism, by promoting osteoblast differentiation and inhibiting osteoclastogenesis, positions it as an agent capable not only of controlling inflammation but also of potentially mitigating structural damage. This distinctive pharmacological profile provides a strong rationale for its application across a spectrum of autoimmune rheumatic diseases, including rheumatoid arthritis, Sjögren’s disease, and ankylosing spondylitis. The following sections critically evaluate the clinical evidence, assessing the efficacy, safety profile, and therapeutic role of iguratimod in these conditions.

## METHODOLOGY

A comprehensive narrative literature review was conducted using established bibliographic databases, including PubMed/MEDLINE, Web of Science, Scopus, and DOAJ. These databases were prioritised to ensure high-quality and peer-reviewed scientific evidence. Supplementary searches were performed using Google Scholar solely for cross-referencing purposes.

The literature search included publications from the last 10 years, focusing on clinical trials, observational studies, systematic reviews, and meta-analyses on iguratimod and its role in autoimmune diseases. Search terms included “Iguratimod,” “Rheumatoid Arthritis,” “Sjögren’s disease,” “Ankylosing Spondylitis,” “DMARDs,” “Immunomodulation,” and “Autoimmune Diseases.”

Only English-language articles involving human subjects were included. Articles were screened for relevance, methodological quality, and clinical applicability.

### Clinical and Therapeutic Implications of Iguratimod in Autoimmune Rheumatic Diseases

Rheumatic diseases are common chronic musculoskeletal diseases, and their progressive nature leads to high rates of disability if diagnosed late or treated insufficiently. Despite the morbidity caused by these diseases, much remains to be understood and discovered about their aetiologies, epidemiology, and effective treatments.^3^ Due to the inflammatory nature of these autoimmune diseases, commonly used medications include non-steroidal anti-inflammatory drugs (NSAIDs), glucocorticoids, and DMARDs. However, the issues that we currently face in healthcare regarding the management of rheumatic diseases are that these drugs have several adverse effects, making patients intolerant to them and that the number of medicines available today is still low.^13^

Among all these agents, DMARDs were known to have revolutionised care in rheumatism. Non-biologic agents such as methotrexate and hydroxychloroquine remain the first-line of care for many conditions, including RA and Systemic Lupus Erythematosus (SLE). Biological DMARDs, such as TNF inhibitors, IL-1 antagonists, and IL-6 antagonists, also significantly improved rheumatological care upon their introduction. However, the threat of immunosuppression and increased infection rates are the main reasons to use these medications judiciously.^15^ Moreover, the costs imposed on patients by biological agents are pretty high, significantly impacting their treatment, especially in developing countries such as India.^16^ In this era, physicians must have various drug options for medicating rheumatic disease patients to help treat them as effectively as possible while avoiding the harm caused by their idiosyncrasies. IGU is a small-molecule compound identified as a DMARD that exhibits dual immunomodulatory and anti-inflammatory properties. The anti-inflammatory property is due to its inhibition of nuclear factor kappa-B activation, thereby preventing the action of inflammatory cytokines. On the other hand, immunoglobulin production is inhibited by Iguratimod’s action on B cells, as exhibited by extensive clinical research.^2^ Compared to other immunosuppressive agents, Iguratimod causes fewer adverse effects, as evidenced by trials. This is linked to its milder action on lymphocytes compared to other drugs. Common adverse effects seen were dermatitis, hepatic dysfunction, and gastrointestinal symptoms such as vomiting, loose stools, and abdominal pain.^12,13^ However, prolonged treatment with Iguratimod demonstrated a manageable safety profile and was deemed safe for patients, provided close monitoring of liver enzymes was practised.^7,10,17^ The most common indications for Iguratimod use currently are rheumatoid arthritis, Sjögren’s disease, ankylosing spondylitis, and IgG4-related diseases.^13^

### Rheumatoid Arthritis

Iguratimod has already been proven to be an effective treatment modality for rheumatoid arthritis by many research trials.^6,7^ It was found to have a more rapid onset of effect than methotrexate.^13^ Moreover, treatment combining Iguratimod and methotrexate showed better outcomes than methotrexate alone beyond 24 weeks.^18^ A 24-week trial of Iguratimod’s efficacy with daily clinical use was conducted in Japan and revealed significantly decreased levels of C-reactive protein (CRP), Erythrocyte Sedimentation Rate (ESR), tender joint count (TJC), swollen joint count (SJC), etc. Although Iguratimod monotherapy did not significantly lower MMP-3 levels, combination therapy with Iguratimod and methotrexate resulted in significantly lower MMP-3 levels at 24 weeks ^4^.^4^ Therefore, many trials have demonstrated the efficacy and advantages of the Iguratimod and methotrexate combination therapy over methotrexate monotherapy.^4,11^ Furthermore, the 2015 APLAR guidelines recommend Iguratimod (IGU) as a first-line treatment for rheumatoid arthritis in Asia-Pacific countries, particularly for patients intolerant of methotrexate (MTX). IGU is added after an inadequate response to triple DMARD therapy before escalating to biologics or JAK inhibitors, especially in healthcare settings where such escalation is constrained by affordability or access issues.^6,19^ In the current scenario, where generic JAK inhibitors (JAKi) are available, IGU can be considered as an add-on or complementary treatment in patients who show an inadequate response to JAKi therapy, rather than replacing a DMARD initially.^20^

The efficacies of Iguratimod and salazosulphapyridine were also compared in a trial done by Hara et al., which revealed that both drugs had equal efficacy. Moreover, there was no statistical difference in the adverse effects observed with both drugs, but their safety profiles differed.^6^ This explores the possibility of using Iguratimod in patients intolerant to salazosulphapyridine. It is a valuable drug for the management of patients who are unresponsive to other DMARDs. The long-lasting efficacy and safety of Iguratimod make it a valuable treatment option for RA.

While combination therapy for the treatment of RA is a popular choice, rheumatologists often have to be cautious when employing DMARDs due to the numerous adverse effects seen in patients. A standard dual therapy employed for RA is a combination of methotrexate and leflunomide. However, leflunomide has several harmful effects, such as hepatotoxicity, hematopoietic toxicity, and gastrointestinal disturbances.^8^ This could make it an unsuitable choice for patients intolerant to these effects. A multicentre, randomised, double-blind, double-dummy, controlled clinical trial by Tian X et al. studied the comparison between patients receiving methotrexate and IGU (group A) with patients receiving methotrexate and leflunomide (group B). It revealed that, while there was no statistically significant difference in ACR20 achievement rates between group A and group B (84.1% versus 81%, respectively), group A reported fewer adverse events.^5^

This further supports the notion that while IGU is comparable in efficacy to most DMARDs currently in regular use for the treatment of RA, it has a much better safety profile. Due to the high incidence of adverse reactions to DMARDs and treatment-resistant rheumatoid arthritis, combination therapy is commonly required in the management of RA.^11^ Hence, treatment, including iguratimod, may be studied as a new management option for such patients.

Clinical studies and retrospective data indicate that a 6-month period is appropriate to evaluate the effectiveness of IGU after initiation or addition to therapy. Disease activity scores (e.g., DAS28-CRP) and clinical disease activity indices showed significant improvement within 6 months of IGU treatment, which can serve as a benchmark for assessing response or failure.^20^

The Randomised Controlled Trials supporting iguratimod’s efficacy in RA are robust in design—typically multicentre, double-blind, and placebo-controlled— several limitations warrant consideration. Trials such as Hara et al. (2007) and Tian et al. (2020) were conducted exclusively in Asian populations, which may limit generalisability to Western cohorts due to potential ethnic differences in drug metabolism or disease phenotype.^5,6^ Furthermore, many studies, including the pivotal trial by Ishiguro et al. (2013), enrolled patients with inadequate responses to methotrexate but did not always account for concomitant steroid use or prior biologic exposure, potentially confounding outcome assessments.^11^ Most trials also employed relatively short follow-up periods (24–52 weeks), leaving long-term efficacy and safety data to be inferred from open-label extensions or post-marketing surveillance.^7^ Despite these limitations, the consistency of positive findings across trials, large sample sizes, and rigorous methodology strengthens the validity of iguratimod’s reported benefits in RA.

### Sjögren’s disease

Sjögren’s disease (SS) is a disorder that affects the body’s exocrine glands, including the salivary glands and pancreas, leading to the drying of mucosal surfaces and further complications. Due to an abnormal immune response to the autoantigens implicated in this disease, i.e. SSA and SSB antigens, the epithelium of these glands is destroyed, resulting in the clinical manifestations seen in Sjogren’s. Increased B-cell activity in SS leads to the production of autoantibodies against these antigens. Moreover, this phenomenon increases the risk of B-cell lymphoma among Sjogren’s patients.^14^ Because Iguratimod can inhibit B-cell activity by modulating the protein kinase C/C/early growth response 1 pathway, this drug has been implicated in changing the treatment landscape for Sjogren’s disease.^21^

An open-label pilot study involving 27 patients with Sjogren’s syndrome, as per the revised American-European Consensus Group criteria, found that IGU administration significantly improved disease manifestations. The EULAR Sjögren’s disease (SjD) disease activity index (ESSDAI) scores were significantly reduced with IGU use, and rheumatoid factor levels decreased considerably as well.^22^ Another study by Q Shao et al. assessed the efficacy of IGU in patients with primary Sjogren’s syndrome and reported successful results. A randomised, single-blinded, placebo-controlled trial was conducted in 66 primary Sjogren patients for 24 weeks, with participants randomised in a 2:1 ratio to receive IGU or placebo. The primary outcome was the EULAR Sjogren’s Syndrome Patient-Reported Index (ESSPRI), which was significantly lower in patients receiving IGU. Other parameters, such as the Mental discomfort VAS score, Patient global assessment, Schirmer’s test, ESR, and IgG, were significantly better in patients on IGU.^23^

Sjogren’s is commonly treated with combination therapy. While these studies have proved the usefulness of IGU in SS as monotherapy, studies have been conducted that proved IGU to be a capable drug in the setting of combination therapy as well. Dual treatment with methylprednisolone and IGU was compared with a combination of methylprednisolone and hydroxychloroquine. Results revealed that combination therapy with methylprednisolone and IGU showed an effective rate of 93.6%, whereas the traditional combination of methylprednisolone and hydroxychloroquine showed an effective rate of only 76.6%.^24^ These studies suggest that IGU could be a highly effective medication for treating SS, and further trials and research will help define better protocols to manage Sjogren’s syndrome.

### Ankylosing spondylitis

Ankylosing spondylitis belongs to a category of rheumatic diseases known as spondyloarthritides. It is marked by symptoms such as inflammatory back pain, asymmetrical peripheral oligoarthritis, and involvement of particular organs, including anterior uveitis, chronic inflammatory bowel disease, and aortic root involvement. AS is typically treated with NSAIDs to control pain and stiffness. DMARDs such as infliximab and adalimumab have also demonstrated efficacy in managing AS, as shown by multiple large, randomised, placebo-controlled trials. However, these drugs can often have serious consequences for the patients. NSAIDs are known to cause cardiovascular, gastrointestinal, and renal adverse effects when used chronically.^25^ TNF blockers are expensive medications, and patients may be intolerant of them due to the adverse impacts, such as increased rates of respiratory tract infections and hepatotoxicity.^26,27^ A study by LX Pang et al. assessed the effects of IGU in patients with AS. The control group received etanercept; accordingly, the observation group received oral IGU. Parameters such as ESR, Bath Ankylosing Spondylitis Disease Activity Index (BASDAI), serum TNF-alpha, and CRP were all significantly improved in the observation group (p<0.05).^10^ A study was conducted to study the effect of IGU on patients with active spondyloarthritis. It was found that patients responded well to IGU, with higher percentages of responders to the Assessment in Ankylosing Spondylitis (ASAS) 20% (ASAS20) and 40% (ASAS40) criteria. Moreover, there was no statistically significant difference (p<0.05) in the adverse effects in both groups.^28^ Numerous additional studies have demonstrated the effectiveness of IGU in treating AS.^10^ Therefore, Iguratimod should be investigated as a potential new treatment for patients with ankylosing spondylitis, as it could significantly improve their quality of life. Several other studies are currently underway to evaluate the possible use of Iguratimod in various rheumatic diseases. The usage of IGU in lupus nephritis, IgG4-related diseases, and kidney transplantation is presently being studied, primarily in hospitals in China.^13^

The clinical application of Iguratimod has been extensively studied for some rheumatic diseases, while others still require further trials to establish its position as a new, practical treatment option. Our literature review revealed that most recent studies have been in China. The absence of Indian or Western cohort studies limits the generalisability of current findings, highlighting a critical gap for future pharmacogenomic and efficacy research. Trials must be conducted in India to assess any variations in Iguratimod potency due to geographical and racial differences compared to the Eastern population. However, we hope that Iguratimod becomes a more popular option for treating rheumatological diseases in India as well, as it has the potential to change the landscape for managing multiple immune diseases.

### Geographic and Ethnic Gaps

A salient limitation in the current literature on iguratimod is its exclusive reliance on East Asian populations. All pivotal randomised controlled trials, long-term extension studies, and the comprehensive post-marketing surveillance data, including the seminal 52-week safety report by Mimori et al. involving 2,666 Japanese patients, originate from Japan and China.^7,29^ To our knowledge, no dedicated clinical trials have been conducted in Western (European or North American) cohorts, nor in other diverse ethnic populations such as those in South Asia or Africa.

This geographic homogeneity raises critical questions regarding the generalisability of findings. Potential ethnic and pharmacogenetic variability could influence iguratimod’s pharmacokinetics, efficacy, and safety profile. Differences in allele frequencies of HLA-DRB1 shared epitope genes, polymorphisms in cytokine genes (e.g., TNF-α, IL-6), or variations in drug-metabolising enzymes (e.g., CYP2C9, CYP3A4) may lead to divergent treatment responses and adverse event rates between populations.^30^ For instance, the higher prevalence of specific HLA-DRB1*04 subtypes in Caucasian RA patients is associated with differential responses to other DMARDs; whether this affects iguratimod response is unknown.^31^ While preliminary investigator-initiated studies have been registered in the United States, results are not yet available.

The implication for global practice is significant. Clinicians outside East Asia must extrapolate data without clear evidence of external validity. This underscores an urgent need for prospective, multicentre trials in Western and other ethnic populations, as well as pharmacogenomic studies to identify biomarkers of response. Until such data are available, the application of iguratimod in non-Asian patients should be approached with cautious optimism and rigorous post-marketing surveillance.

### Safety and Tolerability Profile

The post-marketing study conducted in Japan showed that the clinical safety of long-term IGU use was satisfactory. The incidences of AEs, ADRs, serious AEs, and severe ADRs were 46.92% (1251/2666), 38.26% (1020/2666), 7.35% (196/2666), and 4.58% (122/2666), respectively. The most frequent ADRs reported in the study were hepatic function abnormality (5.06%), stomatitis (2.59%), and alanine aminotransferase increase (1.80%). Severe ADRs were pneumonia (0.83%), inter-stitial lung disease (0.60%), and *Pneumocystis jirovecii* pneumonia (0.30%).^29^

The study conducted by Mu et al. confirmed the effectiveness and safety of IGU as a new DMARD. However, the study found that 51.7% of patients (906/1751) experienced at least one adverse event. The most common adverse events included infections (0.6%), abdominal discomfort (0.5%), fractures (0.4%), and increased alanine aminotransferase (0.2%). No deaths were attributed to the use of IGU.^32,33^ The Summary of Clinical Studies on Iguratimod in Autoimmune Diseases is given in **Table 1.**

**Table 1.** Demographic characteristics of the participants (n = 126).

**Disease**	**Author(s) & Year**	**Study Type**	**Sample Size**	**Comparator/Intervention**	**Key Findings**
**Rheumatoid Arthritis**	Hara et al., 2007^6^	RCT, double-blind, multicentre	~376	IGU vs SASP vs placebo	IGU showed efficacy similar to that of SASP, with a favourable safety profile.
	Ishiguro et al., 2013^11^	RCT, double-blind, placebo-controlled	376	IGU + MTX vs MTX + placebo	IGU + MTX significantly improved ACR responses in MTX-inadequate responders.
	Tanaka et al., 2015^17^	Review/Clinical observation	—	IGU in daily clinical practice	Supported efficacy and safety of IGU for long-term RA treatment in Japan.
	Mimori et al., 2017; 2019^7^	Post-marketing surveillance (Japan)	2666	IGU monotherapy	Long-term IGU use was well tolerated; no deaths attributed to IGU; most common ADRs were hepatic-related.
	Tian et al., 2020^5^	RCT, double-dummy, multicentre	450	IGU + MTX vs LEF + MTX	Comparable efficacy, but IGU had fewer adverse events than LEF.
	Duan et al., 2015^18^	RCT	~160	IGU + MTX vs MTX monotherapy	The combination group showed improved outcomes and was well-tolerated.
	Ebina et al., 2023^20^	Cohort study (ANSWER study)	—	Add-on IGU vs JAKi	IGU improved outcomes when added after JAKi failure.
**Sjögren’s disease**	Chen et al., 2021^22^	Open-label pilot study	27	IGU monotherapy	Reduced ESSDAI, IgG, and RF levels; improved symptoms.
	Shao et al., 2021^23^	RCT, single-blind, placebo-controlled	66	IGU vs placebo	Significantly better ESSPRI, Schirmer's test, ESR, and IgG in the IGU group.
	Xu et al., 2017^24^	Comparative study	90+	IGU + MP vs HCQ + MP	IGU combo is more effective than HCQ combo.
	Jiang et al., 2020^21^	Mechanism + clinical outcome study	—	IGU in SjD	Proposed mechanism: PKC/EGR1 pathway modulation; clinical improvements reported.
**Ankylosing Spondylitis**	Pang et al., 2020^10^	Controlled clinical study	90	IGU + etanercept vs etanercept	Improved BASDAI, CRP, ESR, and TNF-α in the IGU group.
	Li et al., 2021^28^	RCT, double-blind, placebo-controlled	100	IGU vs placebo	Significant ASAS20/40 response rates with IGU; no major safety concerns.
	Long et al., 2023^10^	Meta-analysis	—	IGU in multiple RCTs	Confirmed overall efficacy and safety in AS treatment.

### Dose Optimisation and Safety Management

The established dosing regimen for iguratimod involves a staged escalation to optimise tolerability while achieving therapeutic efficacy. Treatment is initiated at 25 mg once daily for the first four weeks, followed by an increase to the maintenance dose of 25 mg twice daily (50 mg/day) if well-tolerated. This strategy is based on Phase I pharmacokinetic studies, which identified 50 mg/day as the maximum tolerated dose, with higher doses correlating with a disproportionate increase in gastrointestinal and hepatic adverse events.^34^

Dose-response relationships are evident in both efficacy and safety outcomes. While the higher 50 mg/day dose is associated with greater and more rapid improvements in disease activity scores (ACR20/50, DAS28), it also correlates with an increased incidence of adverse drug reactions (ADRs). The most common dose-dependent ADRs include hepatic function abnormalities (elevated ALT/AST, incidence ~5–6%) and gastrointestinal symptoms (abdominal discomfort, nausea, diarrhoea, incidence ~2–5%).^7,29,32^ These are typically mild to moderate, reversible, and manageable with temporary dose reduction or supportive care. Serious but rare adverse events, such as interstitial lung disease (ILD) and *Pneumocystis jirovecii* pneumonia (PJP), have been reported at an incidence of approximately 0.6% and 0.3%, respectively, in post-marketing studies.^29^ Although a strict dose relationship for these events is less clear, they are more frequently reported in patients on long-term, higher-dose therapy, particularly those with pre-existing pulmonary risk factors. Therefore, proactive risk mitigation is paramount. We recommend:

Baseline Assessment: Evaluate hepatic and renal function, and screen for latent respiratory infections.

Strict Adherence to Dose Escalation: Avoid initiating therapy at the full 50 mg/day dose.

Regular Monitoring: Perform liver function tests (LFTs) monthly for the first 3–6 months, then periodically thereafter.

Patient Education: Counsel patients to promptly report symptoms such as dyspnoea, persistent cough, fever, or jaundice.

This vigilant, dose-aware approach allows clinicians to leverage iguratimod’s favourable efficacy, particularly its ACR response rate gains, while minimising its dose-dependent risks, solidifying its position as a safer alternative to agents like leflunomide for MTX-intolerant or combination-therapy candidates.^35^

## CONCLUSION

Iguratimod has demonstrated significant effectiveness and tolerability as a therapeutic option for rheumatoid arthritis, Sjögren’s disease, and ankylosing spondylitis. Its dual immunomodulatory and anti-inflammatory mechanisms—including inhibition of NF-κB, cytokine suppression, and B-cell modulation—offer a distinct pharmacologic profile among synthetic DMARDs. Clinical evidence supports its use both as monotherapy and in combination regimens, with a safety profile that compares favourably with that of agents such as leflunomide.

This review consolidates iguratimod’s established role in RA while delineating its expanding promise in SjD and AS, thereby offering a broader, pan-rheumatic perspective than previous MJR reports. It also candidly addresses the drug’s principal limitation—the exclusive reliance on Asian clinical data—and outlines the implied risks of ethnic variability, underscoring the need for urgent international collaborative research. Furthermore, by detailing the dose–efficacy–safety continuum, this work provides a practical framework for optimising clinical use, positioning iguratimod as a valuable, well-tolerated option within the DMARD armamentarium, especially for patients who are failing or intolerant to conventional therapies.

Future efforts must focus on conducting rigorous Western trials, exploring pharmacogenomic predictors of response, and investigating iguratimod’s efficacy in novel autoimmune indications to fully realise its global therapeutic potential. As evidence continues to accumulate, iguratimod is poised to become an increasingly important tool in the personalised management of autoimmune rheumatic diseases worldwide.
